# Romanian Wormwood (*Artemisia absinthium* L.): Physicochemical and Nutraceutical Screening

**DOI:** 10.3390/molecules24173087

**Published:** 2019-08-25

**Authors:** Elena-Alina Moacă, Ioana Zinuca Pavel, Corina Danciu, Zorin Crăiniceanu, Daliana Minda, Florina Ardelean, Diana Simona Antal, Roxana Ghiulai, Andreea Cioca, Mihnea Derban, Sebastian Simu, Raul Chioibaş, Camelia Szuhanek, Cristina-Adriana Dehelean

**Affiliations:** 1 Department of Toxicology and Drug Industry, Faculty of Pharmacy, “Victor Babes” University of Medicine and Pharmacy, 2 Eftimie Murgu Square, 300041 Timisoara, Romania; 2 Department of Pharmacognosy, Faculty of Pharmacy, “Victor Babes” University of Medicine and Pharmacy, 2 Eftimie Murgu Square, 300041 Timisoara, Romania; 3 Department of Plastic and Reconstructive Surgery, Faculty of Medicine, “Victor Babes” University of Medicine and Pharmacy, 2 Eftimie Murgu Square, 300041 Timisoara, Romania; 4 Department of Botany, Faculty of Pharmacy, “Victor Babes” University of Medicine and Pharmacy, 2 Eftimie Murgu Square, 300041 Timisoara, Romania; 5 Department of Pharmaceutical Chemistry, Faculty of Pharmacy, “Victor Babes” University of Medicine and Pharmacy, 2 Eftimie Murgu Square, 300041 Timisoara, Romania; 6 Department of Pathology, CFR Clinical Hospital, 13-15, Tudor Vladimirescu, 300173 Timisoara, Romania; 7 Department of Physical Chemistry, Faculty of Pharmacy, “Victor Babes” University of Medicine and Pharmacy, 2 Eftimie Murgu Square, 300041 Timisoara, Romania; 8 CBS Medcom Hospital, 12 Popa Sapca Street, 300047 Timisoara, Romania; 9 Department of Orthodontrics, Faculty of Dentistry, “Victor Babes” University of Medicine and Pharmacy, 2 Eftimie Murgu Square, 300041, Timisoara, Romania

**Keywords:** *Artemisia absinthium* L., antioxidants, total phenolic content, cytotoxicity, melanoma and breast cancer cell line, HaCaT cells, inflammation

## Abstract

*Artemisia* species are used worldwide for their antioxidant, antimicrobial and anti-inflammatory properties. This research was designed to investigate the phytochemical profile of two ethanolic extracts obtained from leaves and stems of *A. absinthium* L. as well as the biological potential (antioxidant activity, cytotoxic, anti-migratory and anti-inflammatory properties). Both plant materials showed quite similar thermogravimetric, FT-IR phenolic profile (high chlorogenic acid) with mild antioxidant capacity [ascorbic acid (0.02–0.1) > leaves (0.1–2.0) > stem (0.1–2.0)]. Alcoholic extracts from these plant materials showed a cytotoxic effect against A375 (melanoma) and MCF7 (breast adenocarcinoma) and affected less the non-malignant HaCaT cells (human keratinocytes) at 72 h post-stimulation and this same trend was observed in the anti-migratory (A375, MCF7 > HaCat) assay. Lastly, extracts ameliorated the pro-inflammatory effect of TPA (12-*O*-tetradecanoylphorbol-13-acetate) in mice ears, characterized by a diffuse neutrophil distribution with no exocytosis or micro-abscesses.

## 1. Introduction

Common wormwood (*Artemisia absinthium* L., Asteraceae) is a woody-based perennial herb which grows widely in dry, sunny regions of Eurasia, Northern Africa, North and South America [1]. It may be recognized by its silver-grey leaves, which have a soft silk texture. The upper parts of the plant have a distinctive aromatic scent when bruised. From a biochemical point of view, wormwood stands out due to the synthesis of bitter-tasting metabolites and essential oils [2]. Bitterness is conferred by sesquiterpenes (0.15–0.4%), including absinthin, artabsin, anabsinthin, and matricin [3]. Essential oils are secreted by numerous glandular hairs and secretory ducts [4], providing a content of 2–6 mL/kg volatiles in the dried herb [5]. Several chemotypes of volatile oils are known from different parts of the world according to the main constituent: α-and β-thujone, *cis*-epoxyocimene, *trans*-sabinyl acetate and chrysanthenyl acetate [6]. Other secondary metabolites in wormwood include flavonoids (myricetin, quercetin, rutin, hesperidin), hydroxybenzoic acids (salicylic acid, gallic acid), hydroxycinnamic acids (caffeic acids, coumaric acids, ferulic acid), resveratrol, and other [7].

Wormwood leaves and stems have traditionally been employed as a bitter tonic in appetite loss. Preparations are also prescribed as a choleretic in dyspeptic disorders, and in liver diseases due to its hepatoprotective effect. The herb is as well known for its anthelmintic, antipyretic, antibacterial and insecticide properties [5]. The importance of the herb is acknowledged by its inclusion in the European Pharmacopoeia and other official compendia, as well as by the monograph of the Committee on Herbal Medicinal Products of the European Medicine Agency (EMA/HMPC/751484/ 2016).

In recent years, wormwood has attracted considerable attention due to its anti-inflammatory effect. Clinical studies could point out favorable effects in Crohn’s disease. Patients receiving an herbal blend containing wormwood as an add-up to standard steroids could afford the lowering of steroid doses; the remission of the disease could be pointed out in 65% of the patients after eight weeks of treatment [8]. The favorable evolution was explained by the suppression of tumor necrosis factor alpha (TNF-α) by some metabolites from *A. absinthium* extracts, and the potential of wormwood to treat diseases with an augmented production of pro-inflammatory cytokines was pointed out [9]. Wormwood extracts proved as well efficient in the supportive treatment of early-stage IgA nephropathy [10]. 

The current research aims to investigate in depth both the chemical composition as well as the biological activity of ethanolic extracts obtained from leaves and stems of wormwood collected from the Southern part of Romania in order to enhance the knowledge about the phytochemistry and bioactivity of this ancient medicinal plant with important medicinal potential. To this end, ethanol extracts of leaves and stems were investigated by liquid chromatography–mass spectrometry (LC-MS), thermal analysis (TG-DSC) and Fourier-transform infrared spectroscopy (FT-IR). The Total Phenolic Content (TPC) was evaluated spectrophotometrically, and radical scavenging effects as well as the cytotoxic and anti-migratory potential on two tumor cell lines–melanoma and breast adenocarcinoma cells and on a non-tumor cell line–HaCaT–keratinocytes was assessed. The anti-inflammatory effects of the extracts were tested in a mouse ear edema model.

## 2. Results

### 2.1. Physicochemical Analysis

After lyophilization, both ethanolic extracts, obtained from leaves and stems of wormwood were characterized by thermal analysis (TG-DSC) and Fourier-transform infrared spectroscopy (FT-IR).

#### 2.1.1. Thermal Analysis

**Figure 1:**
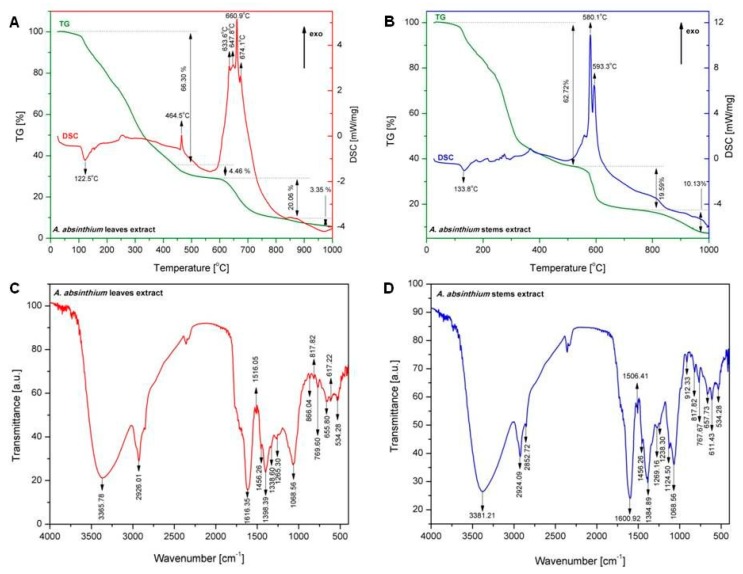
**A**—TG-DSC curves of *A. absinthium* leaves extract, **B**—TG-DSC curves of *A. absinthium* stems extract, **C**—FT-IR spectra of *A. absinthium* leaves extract, **D**—FT-IR spectra of *A. absinthium* stems extract.

TG-DSC curves of dried and lyophilized extracts of *A. absinthium* L. leaves and stems are depicted in Figure 1A,B. Thermal analysis performed for dried and lyophilized wormwood leaves extract revealed a total mass loss of 94.17% in four stages. In the first stage, the wormwood leaves extract loses 66.30% of the total mass and an endothermic process which can be noticed with a maximum at 122.5 °C. The second stage is located between 450 °C and 600 °C; within this interval the sample loses 4.46% of the total mass and shows an exothermic effect with a maximum at 464.5 °C. In the third stage of extract degradation, four exothermic processes occur between 600 °C and 700 °C, and a total mass loss of 20.06% was measured. In the final stage of extract degradation only a slight mass loss (3.35%) without any thermal effect could be determined.

Thermal analysis performed for dried and lyophilized wormwood stems extract revealed a total mass loss of 92.44% in three stages. The largest mass loss (about 62.72%) occurs during the first stage; it is accompanied by an endothermic process with a maximum at 133.8 °C. In the second stage (temperature range 500 °C–650 °C) a mass loss of 19.59% was registered, related to two exothermic processes peaking at 580.1 °C and 593.3 °C, respectively. In the final stage of the extract degradation only a mass loss of 10.13% occurred.

#### 2.1.2. FT-IR Investigations

In order to identify the functional groups of the active components which are present in the leaves and stems extracts of *A. absinthium*, the FT-IR analysis was done. The FT-IR analysis is based on the presence of the peak values in the region of IR radiation appeared on different wavenumber. The results of analysis of dried lyophilized leaves and stems extracts based on *A. absinthium* are given in Table 1 and Figure 1C,D. The two lyophilized extracts based on *A. absinthium* exhibited a similar IR profile most probably due to the polar nature of molecules present in both extracts.

### 2.2. Phenolic Composition

#### 2.2.1. Raw Profiling

The results of our study indicated a higher total phenolic and flavonoid content in the extract obtained from wormwood leaves as compared to the stems extract (Table 2).

#### 2.2.2. LC-MS Fingerprint

In Table 3 are presented the polyphenolic content of the *A. absinthium* L. leaves and stems, obtained by LC-MS analysis.

#### 2.2.3. Antioxidant Activity

**Figure 2:**
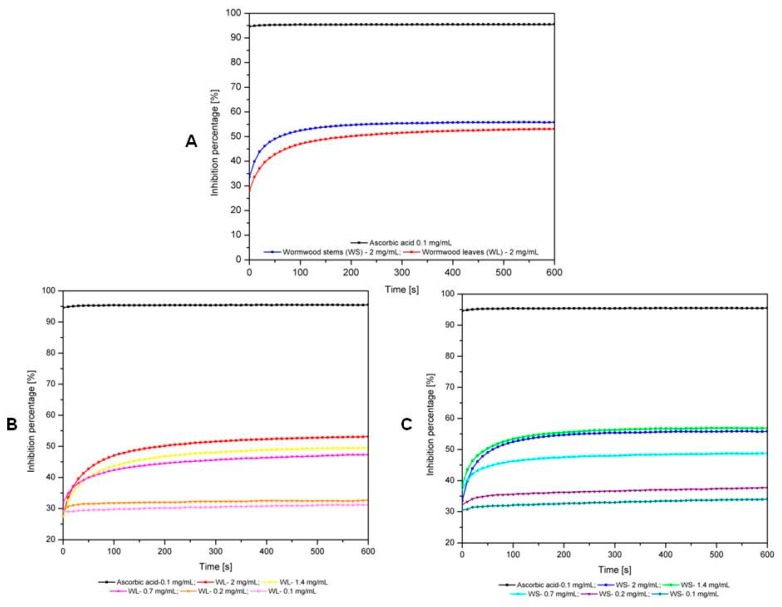
The time dependent inhibition percentage of *A. absinthium* extracts: **A**—*A. absinthium* stock solutions based on leaves and stems vs. ascorbic acid; **B**—extracts of *A. absinthium* leaves vs. ascorbic acid; **C**—extracts of *A. absinthium* stems vs. ascorbic acid.

In order to evaluate the antioxidant activity (AOA), both wormwood lyophilized extracts were dissolved in ultra-pure distilled water. The values corresponding to the inhibition percentages of the two extracts of *A. absinthium* (leaves and stems) were evaluated in time, and compared to those of ascorbic acid solution (control) are presented in Figure 2A. As it can be noticed from the graph, both extracts of *A. absinthium* show antioxidant effects during the time frame of the evaluation. Moreover, the AOA values of *A. absinthium* stem extract are slightly higher than the AOA values of *A. absinthium* leaves extract.

The reduction rate of DPPH free radical is different for the two extracts. The wormwood stems extract quenches the free radicals after 250 s; subsequently the reaction reaches equilibrium. Wormwood leaves extract quenches the entirety of free radicals after 500 s.

From Figure 2B it can be noted that the antioxidant activity of *A. absinthium* leaves extract is concentration-dependent. In the case of *A. absinthium* stems extracts the values corresponding to inhibition percentages are not directly proportional with their concentration; the maximal inhibition was measured for the sample containing 1.4 mg extract/mL.

Table 4 presents the percent inhibition of leaves and stems extracts. The values were calculated with equation (1) and subsequently used for the determination of IC_50_ values. IC_50_, characterizing the antioxidant activity as evaluated by the DPPH test, were as follows: for ascorbic acid IC_50_ = 0.03191 ± 0.0019 mg/mL; for wormwood leaves extract IC_50_ = 0.4993 ± 0.0201 mg/mL and for wormwood stems extract IC_50_ = 0.4865 ± 0.0182 mg/mL. The IC_50_ parameter for each sample was determined using GraphPad Prism 8 software. Correlation coefficients were R^2^ = 0.9856 for wormwood leaves extract and R^2^ = 0.9855 for wormwood stems extract.

### 2.3. Bioactivity

#### 2.3.1. Cytotoxicity and Selectivity Index Assessment

The effect of *A. absinthium* ethanolic extracts was evaluated on a non-tumor and two tumor cell lines at different periods of time and compared to the Control group (cells stimulated with ultrapure water). In Supplementary Figure S1 is described the in vitro cytotoxic effect at 24 h and at 48 h post-stimulation. At 24 h post-stimulation, both wormwood leaves and stems extracts showed no cytotoxic effect on the non-tumor cell line, HaCat, regardless of the tested dose. On A375 melanoma cells, *A. absinthium* leaves extract elicited a mild decrease of cell viability at the higher dose tested, 1000 μg/mL (cell viability 86.2 ± 2.2% vs. Control), whereas *A. absinthium* stems extract provoked a slightly more cytotoxic effect at the same dose (cell viability 82.2 ± 4.9% vs. Control). At 24 h, both extracts induced a decrease in MCF7 breast adenocarcinoma cell viability, the most significant results being obtained at the highest tested dose (for *A. absinthium* leaves extract at 1000 μg/mL the cell viability was 84.3 ± 6.1% vs. Control and for the stems extract it was 80.9 ± 3.1% vs. Control).

At 48 h post-stimulation, on HaCaT keratinocytes, wormwood extracts induced a mild decrease in cell viability, the effect being more noticeable in the case of *A. absinthium* leaves extract (at 1000 µg/mL, cells viability was 92.8 ± 4.6% vs. Control). On the tumor cell lines, both wormwood extracts provoked a dose dependent decrease of cells viability, with the best results obtained after stimulation with the stems extract. For A375 cells, at the highest dose, the stems extract decreased the viability to 68.8 ± 4.3% vs. Control, while the same extract induced a viability of 56.9 ± 4.4% in MCF7 cells vs. Control cells. The obtained data shows that the wormwood extracts affect more the cancer cells than the non-tumor cells, especially at higher doses.

**Figure 3:**
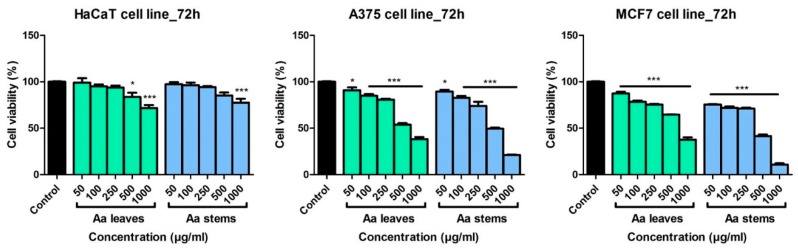
In vitro cytotoxicity assessment of *A. absinthium* leaves and stems ethanolic extracts (50, 100, 250, 500 and 1000 µg/mL) on a non-tumor cell line–HaCaT–human keratinocytes and on two human tumor cell lines A375—melanoma cells and MCF7–breast adenocarcinoma cells at 72 h post-stimulation by the means of Alamar blue assay. The results are expressed as cell viability percentage (%) related to Control (cells stimulated with ultrapure water). The data represent the mean values ± SD of three independent experiments. Comparison among the groups was performed using the One-way ANOVA test followed by Dunnett’s post-test. (* *p* < 0.05; *** *p* < 0.001).

Figure 3 presents the effect of wormwood extracts at 72 h post-stimulation. On HaCaT cells, the extracts induced a decrease in cell viability especially at 1000 µg/mL: cell viability was 71.6 ± 7.3% vs. Control in case of *A. absinthium* leaves extract, and 77.6 ± 9.2% vs. Control in case of *A. absinthium* stems extract. These data show that the leaves extract induced a more pronounced decrease in HaCaT cell viability than wormwood stems extract. Regarding the tumor cell lines, *A. absinthium* extracts elicited a significant cytotoxic effect at all the tested doses. Moreover, *A. absinthium* extracts affect rather the tumor cells than the non-tumor cell line, HaCaT. At 1000 µg/mL, on A375 melanoma cells, *A. absinthium* leaves extract decreased cells viability to 38.2 ± 3.8% vs. Control and *A. absinthium* stems extract to 21.06 ± 1.1% vs. Control. On the breast adenocarcinoma cells, at the same dose, *A. absinthium* leaves extract decreased cells viability to 37.6 ± 4.3% vs. Control and the stems extract to 10.8 ± 2.3% vs. Control. For both tumor cell lines, the most potent cytotoxic effect was obtained in the case of the stems extracts and the highest reduction of tumor cells viability was noticed for MCF7 breast cancer cells.

The IC_50_ values for the tumor and non-tumor cells following 72 h stimulation with *A. absinthium* extracts are shown in Table 5. The highest IC_50_ value was recorded in the case of HaCaT cells stimulated with *A. absinthium* leaves extract, whereas the lowest values were obtained for the breast cancer cells, MCF7. These data indicate that the tumor cell lines are more affected than the non-cancer cells following stimulation with wormwood extracts.

Within this study the Selectivity index (SI) of *A. absinthium* leaves and stems extracts was also determined. The non-malignant HaCaT cells, human keratinocytes, were used as a control in order to evaluate the degree of selectivity of wormwood extracts for the tumor cell lines. The values obtained for the SI range between 1.16 (for A375 cells stimulated 72 h with *A. absinthium* stems extract) and 1.59 (for MCF7 cells stimulated 72 h with *A. absinthium* leaves extract), showing a slightly more active effect of wormwood on the breast adenocarcinoma cells. Following the calculation of SI we can indicate that the extracts did not display a significant selective effect towards the cancer cells.

#### 2.3.2. Scratch Assay Assessment

In order to determine the anti-migratory potential of *A. absinthium* extracts a scratch assay technique was performed. The non-tumor and tumor cell lines were stimulated with different concentrations of wormwood extracts and compared to Control (cells stimulated with ultrapure water).

**Figure 4:**
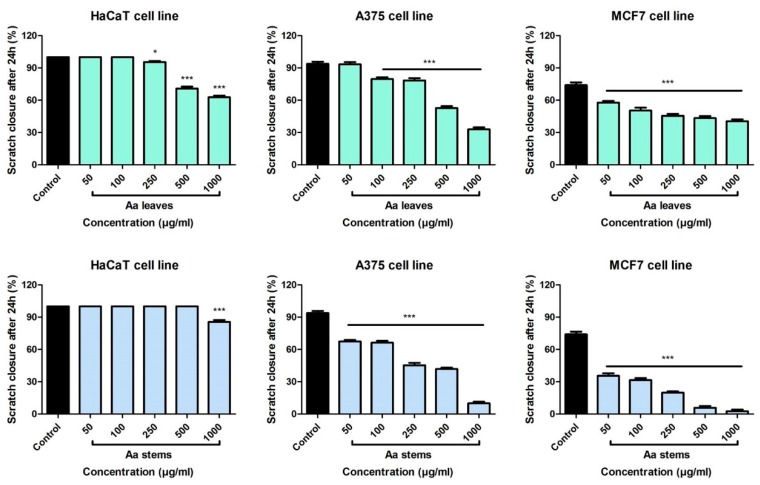
The migratory potential of HaCat, A375 and MCF7 cells following treatment with *A. absinthium* leaves and stems ethanolic extract (50, 100, 250, 500 and 1000 µg/mL). Images were taken by light microscopy at 10× magnification (the scale bars represent 100 μm). The bar graphs are expressed as percentage of scratch closure after 24 h compared to the initial surface. Comparison among the groups was performed using the One-way ANOVA test followed by Dunnett’s post-test. (* *p* < 0.05; *** *p* < 0.001 vs. Control-cells stimulated with ultrapure water).

In Figure 4, the effect of *A. absinthium* leaves and stems extracts on HaCaT, A375 and MCF7 cells is shown. Regarding the effect of *A. absinthium* leaves on HaCaT cells, at low concentrations (50 and 100 µg/mL) stimulation with the extract induced a pro-migratory effect upon the non-tumor cells, the results being similar to those obtained in the Control group. For these concentrations the scratch closure rate after 24 h was 100%. At the concentration of 250 µg/mL, the leaves extract elicited a mild anti-migratory effect, showing a scratch closure rate of 95.4%. On HaCaT cells, at the higher doses tested, *A. absinthium* leaves extract slowed down the migratory effect, showing a scratch closure of 70.8% (500 µg/mL) and 62.7% (1000 µg/mL), respectively. Stimulation with the stems extract elicited a significant pro-migratory effect on the HaCaT cells with scratch closure rate of 100% for concentration ranging from 50 to 500 µg/mL. Only at the highest dose tested, an anti-migratory effect was noticed, having a scratch closure percentage of 85.5%. The data obtained indicate that the stems extract exhibited a superior pro-migratory effect on the non-tumor cells. Furthermore, no changes in the shape and morphology of HaCaT cells were noticed following stimulation with the samples (see Supplementary Figure S2).

Stimulation of human A375 melanoma cells with *A. absinthium* leaves extract proved to have a dose dependent anti-migratory effect (Figure 4). At the lowest tested dose, 50 µg/mL, the leaves extract provoked a closure of the scratch of 93.3%, similar to the Control group, while at the highest tested dose, 1000 µg/mL, the closure was 32.9%. Moreover, at 24 h post-stimulation, it can be seen in the pictures that at the highest concentration there is a slightly change in the cells shape, some of them displayed round shape, indicating that the leaves extract affects melanoma cells (see Supplementary Figure S2).

*A. absinthium* stems extract manifested a significant anti-migratory effect on A375 cells at all the tested concentrations. The results show that the stems extract had a stronger inhibitory effect on the migration of melanoma cells. At the highest tested dose, the scratch closure rate was around 9.9%. Furthermore, at this concentration, cells with round shape and detached ones were present, showing the cytotoxic effect of this extract. The results obtained are consistent with the ones from the cell viability assay, proving an anti-migratory and cytotoxic effect of these extracts.

Regarding the anti-migratory effect of *A. absinthium* leaves extract on MCF7 human breast adenocarcinoma cells, at 24 h post-stimulation, all the evaluated concentrations manifested a significant inhibitory effect on cancer cells migration.

The anti-migratory properties on MCF7 cells were more potent after stimulation with *A. absinthium* stems extract compared to the effects obtained in the case of the leaves extract. Application of the stems extract provoked at the concentration of 500 µg/mL a closure percentage of 5.8% and for 1000 µg/mL it was 2.6%. Furthermore, it can be seen in the images from Supplementary Figure S2, that at 500 and 1000 µg/mL, the cells had a modified morphology with round shape and also many cells are detached from the plate (Supplementary Figure S2). These elements suggest that the stems extract had a cytotoxic effect on the breast adenocarcinoma cells.

#### 2.3.3. Anti-Inflammatory Assessment

**Figure 5:**
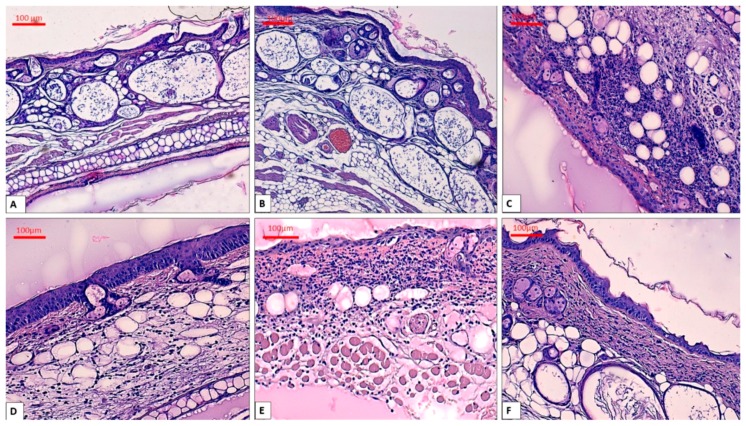
Histological aspects of the skin, H&E stain, **A**: Control group—with no intervention, magnification ×10; **B**: Acetone group showing edema, magnification ×10; **C**: TPA group showing abundant inflammation in the entire dermis with abscess formation, magnification ×20; **D**: TPA + indomethacin group depicting mild inflammation, magnification ×20; **E**: topical application of *A. absinthium* leaves extract indicating moderate inflammation and moderate interstitial edema, magnification ×20; **F**: treatment with *A. absinthium* stems extract having a reduced number of neutrophils and thick collagen fibers in dermis, magnification ×20.

Skin specimens from the control group had normal histological structures, while the group treated with acetone showed marked edema and congestion of the blood vessels but without inflammation or changes of the epidermal thickness (Figure 5A,B).

As illustrated in Figure 5C, TPA treatment produced a marked pro-inflammatory action on mice ears. The epidermal thickness was increased and it was accompanied by reactive changes of the cells. We noticed an abundant inflammatory infiltrate composed of neutrophils, disposed in the papillary and reticular dermis and around the dermal appendages. The inflammation showed a diffuse pattern, but it also formed micro-abscesses. In some areas, neutrophils migrated in the epidermal thickness leading to exocytosis. In addition, the blood vessels were congested and some of them displayed leukocytes margination. Intraepidermal and dermal edema was noted in this group.

Topical administration of indomethacin (Figure 5D) provoked a reduction of the inflammatory processes compared to the TPA group. The inflammatory infiltrate was composed of a reduced number of neutrophils that were diffusely disposed in the dermis. Exocytosis and the abcesses were absent in this group and dermal edema was mild.

In the group treated with *A. absinthium* leaves extract (Figure 5E) the epidermis was focally enlarged compared to the untreated group. The inflammation was moderate with a diffuse distribution of the neutrophils in the dermis. No exocytosis or micro-abscesses were observed in this group. Congestion of the blood vessels and interstitial edema were mild and some blood vessels showed leucocytes margination.

In contrast, topical application of the *A. absinthium* stems extract (Figure 5F) showed a clearer anti-inflammatory effect. In this group, the inflammatory infiltrate was mild and diffusely distributed in the dermis and moderate in only few areas. The interstitial edema was mild and focal, while congestion and leucocytes margination of the blood vessels were absent. Moreover, the dermis showed thick collagen fibers organized in compact bundles.

## 3. Discussion

### 3.1. Physicochemical Analysis

Thermal analysis is an analytical method used also to characterize the compounds from plant medicine. Figure 1A,B present the TG and DSC signals obtained during thermal decomposition of, lyophilized extracts based on wormwood leaves and stems, in air. In both extracts, there was a substantial mass loss in the first stage of degradation (above 60%). This step was accompanied by an endothermic effect, occurring at: 122.5 °C in the case of wormwood leaves extract and 133.8 °C in the case of wormwood stems extract. We can assume that this endothermic process is in fact an overlap of endothermic effects associated with water elimination and/or partial decomposition of the two extracts. On the TG-DSC curve of wormwood leaves extract, the oxidative degradation occurs with the formation of several exothermic peaks between 464.5 °C and 674.1 °C. The exothermic effect recorded at 464.5 °C, associated with a lower mass loss (4.46%), can be related to the degradation of aromatic amino-acids and/or carbohydrates present in the leaves extract. Continuous exothermic effects can be observed on DSC curve between 633.6 °C and 674.1 °C; these effects could be associated with the oxidative degradation of a wide variety of metabolites, principally phenolics, simple amines and several aromatic compounds. The four exothermic processes recorded on the DSC curve, are accompanied by a mass loss of 20.06% recorded on the TG curve. In the final stage of degradation, only a small mass loss of 3.35% can be seen, which can be assigned to the burning of organic residues (carbon). On the TG-DSC curve of wormwood stems extract, the oxidative degradation occurs with the formation of two exothermic peaks at 580.1 °C and at 593.3 °C, associated with a mass loss of 19.59%. These exothermic effects could be related with the degradation of aromatic compounds, carbohydrates and aromatic amino-acids present in the stems extract. 

FT-IR spectroscopy analysis is one of the most widely technique that can be utilized for the qualitative analysis of pharmacologically active compounds from various plant species. The FT-IR spectrum presents unique bands which express the presence of functional groups contained by the molecules in the extracts; it constitutes a chemical fingerprint [11].

Figure 1C,D show that the FT-IR spectra of *A. absinthium* leaves and stems extracts had relevant absorption peaks at 3365.78 cm^−1^ (for leaves) and 3381.21 cm^−1^ (for stems); 2926.01 cm^−1^ (for leaves) and 2924.09 cm^−1^ and 2852.72 cm^−1^ respectively (for stems); 1616.35 cm^−1^ (for leaves) and 1600.92 cm^−1^ (for stems) and at 1068.56 cm^−1^ for both wormwood extracts. The broad bands at 3365.78 (for leaves extract) and 3381.21 cm^−1^ for stems extract correspond both to O-H stretching of hydroxyl groups from alcohols, phenols and carboxylic acids [12] as well as N-H stretching of amines or amides [13]. The peak situated at 2926.01 cm^−1^ for leaves as well as 2924.09 cm^−1^ and 2852.72 cm^−1^ respectively, for stems extract, corresponds to the saturated aliphatic C-H bonds. In the case of stems extract, the two values correspond to C-H stretching which indicate the occurrence of aromatic ring and alkyl group attachment. This may be corresponding to C-C stretching vibration of aromatic amines [14]. Moreover, the band situated at 2926.01 cm^−1^ in case of leaves extract, may correspond to the CH_3_ vibrations which exist in the functional groups of chlorophyll present in wormwood extract [15]. The peak at 1616.35 cm^−1^ in the case of leaves extract and the peak at 1600.92 cm^−1^ showed in the case of stems extract, confirm the presence of amide functional groups, like N-H bending vibration [16]. The analysis of C-H out-of plane bending can often distinguish substitution patterns which may correspond to N-O asymmetric stretch of nitro functional groups, with bands situated at 1516.05 cm^−1^ in the case of leaves extract and 1506.41 cm^−1^ in the case of stems extract. These two bands confirm the presence of aromatic ring [13]. The bands situated at 1398.39 cm^−1^ (for leaves extract) and 1384.89 cm^−1^ (for stems extract) are attributed to the C-H bending vibration of alkanes, which refer to the binding of the aromatic ring –C-H for the in-plane bending absorption. The band situated at 1265.30 cm^−1^ (for the leaves extract) and the band from 1269.16 cm^−1^ as well as 1238.30 cm^−1^ respectively, in the case of stems extract, corresponds to the aromatic acid ester C-O stretching vibration [17]. The bands located in the range 1068.56 cm^−1^ and 1124.50 cm^−1^ can be attributed to C-O stretch vibration that may come from alcohol functional groups (primary, secondary and tertiary) [17]. The bands in the range 534.28 cm^−1^ and 912.33 cm^−1^ represent the out of plane bending vibration from aromatics alkenes =C-H [18]. The FT-IR spectra reveal only the structural information of some functional groups presents in the plant extract and can be used for authentification of some constituents, but completing information with other techniques for determining the quantitative analysis and confirm the presence of pharmacologically active compounds are required.

### 3.2. Phenolic Composition

Phenolic compounds are widely distributed in plants and they are associated with the prevention of several diseases where oxidative stress plays an important role [19]. The total phenolic content of the two wormwood extracts was evaluated due to the importance of this class of compounds for the antioxidant activity. The results showed that the leaves extract have a higher phenolic content (54.68 ± 1.93 mg GAE/g extract) compared to the extract from stems (44.15 ± 1.12 mg GAE/g extract).

The total phenolic content of various wormwood extracts was evaluated in several studies. A higher value was observed for the hydroalcoholic extract obtained from the above ground parts of the plant (9.29 ± 0.51 mg GAE/g dry weight) as compared to the hexane and methanol one [20]. Another study estimated a total phenolic content of 123 ± 0.82 mg GAE/g extract for the methanolic extract obtained from aerial parts of the plant [21].

A variation in the total phenolic content depending on the collecting area was observed. Values of the total phenolic content ranging between 49.39 ± 2.20 mg GAE/g dry weight and 99.89 ± 3.30 mg GAE/g dry weight were determined for methanolic extracts obtained from *A. absinthium* aerial parts [22]. In the plant material collected in Romania, a previous study estimated a total phenolic content of 18.14 mg GAE/g dry weight for aerial parts of the plant [23]. The above ground parts of wormwood collected in Turkey presented a total phenolic content of 9.79 μg GAE/mg [24].

Among polyphenols, flavonoids are known for their antioxidant, anti-inflammatory, antibacterial, antiviral and anticancer properties [25]. The total flavonoid content of wormwood extracts was in agreement with the results obtained for total phenolic content, with a lower value for wormwood stems extract (34.14 ± 2.16 mg CE/g extract) compared to leaves extract (43.08 ± 2.47 mg CE/g extract).

Other studies mentioned higher flavonoid content for the ethanolic extract from wormwood aerial parts as compared to chloroform and aqueous extracts [26]. Variations between 3.02 ± 0.05 and 19.28 ± 0.12 mg quercetin equivalents per gram dry weight were observed for different extracts obtained from the above ground plant material [20]. The highest total flavonoid content estimated for plant material collected in different Tunisian areas was 126.4 ± 2.32 mg catechin equivalent per gram dry weight [22].

Both alcoholic extracts were subjected to LC-MS analysis under identical solution and instrumental conditions which enabled the identification and in some cases quantification, consistent with their Rt and *m*/*z* values, of 9 polyphenolic acids: gentisic acid, chlorogenic acid, caffeic acid, *p*-coumaric acid, isoquercitrin, rutin, quercitrin, luteolin and apigenin expressed in µg/mg d.w. (Table 3). Obtained results indicated that chlorogenic acid was the most abundant polyphenolic quantified compound. Isoquercitrin, rutin and quercitrin were also detected but in smaller concentrations, accompanied by traces of gentisic acid, caffeic acid, *p*-coumaric acid, luteolin and apigenin which were below the limit of quantification. In terms of their concentration in the two types of extract, it seems that *A. absinthium* stems extract are somehow similar to *A. absinthium* leaves extract, *A. absinthium* stems extracts being slyghtly richer, with one exception (Table 3). Thus, chlorogenic acid, isoquercitrin and rutin have concentrations a bit higher in *A. absinthium* stems while quercitrin is more abundant in *A. absinthium* leaves extract (Table 3).

In a similar approach, Ivanescu and co-workers have conducted a HPLC-DAD-MS study in order to determine the polyphenols from the aerial part of three *Artemisia* species, namely *A. absinthium*, *A. annua*, and *A. vulgaris* before as well as after acid hydrolysis. Results for *A. absinthium* have shown that in case of the un-hydrolyzed extract ferulic acid (0.608 mg/100 g dry herb) and kaempferol (2.456 mg/100 g dry herb) could be identified. On the other hand in case of the hydrolyzed extract *p*-Coumaric acid (12.6 mg/100 g dry herb), ferulic acid (2.432 mg/100 g dry herb), fisetin (0.792 mg/100 g dry herb) and patuletin (0.616 mg/100 g dry herb) could be detected [27]. Also Craciunescu and co-workers, used maceration as a method of extraction but ethanol 70% as solvent. Among the phenolic and flavonoid screened compounds they have detected gallic acid (0.092 ± 0.005 mg/g dry extract), chlorogenic acid (0.077 ± 0.004 mg/g dry extract), caffeic acid (0.181 ± 0.009 mg/g dry extract), coumaric acid (0.112 ± 0.006 mg/g dry extract), ferulic acid (0.100 ± 0.005 mg/g dry extract), rutin (0.089 ± 0.005 mg/g dry extract), luteolin (0.677 ± 0.036 mg/g dry extract), quercetin (2.707 ± 0.135 mg/g dry extract), myricetin (0.201 ± 0.011 mg/g dry extract), apigenin (0.359 ± 0.019 mg/g dry extract) [28]. In a comprehensive study conducted by Aberham and co-workers, sesquiterpene lactones, flavonoids and lignans were detected from an extract of *A. absinthium* obtained by maceration with dichloromethane by HPLC-MS [29]. Sahin and co-workers have tried to increase the amount of bioactive compounds from *A. absinthium* by optimization of ultrasonic-assisted extraction. HPLC-DAD analysis has shown the presence the following polyphenols: protocatechuic acid (0.10 ± 0.01 mg/g dried plant), chlorogenic acid (5.72 ± 0.11 mg/g dried plant), caffeic acid (2.07 ± 0.01 mg/g dried plant), ferulic acid (19.57 ± 0.44 mg/g dried plant), rosmarinic acid (7.82 ± 0.01 mg/g dried plant) [30].

For the prevention of various types of cell damage, antioxidants are widely employed compounds. Antioxidants are natural products with the ability to eliminate reactive oxygen species (ROS) in enzymatic and nonenzymatic processes. Plants contain potent antioxidants which act as radical scavengers and mitigate the damaging effects of ROS to the human body [31].

Regarding the antioxidant activity presented in this study, it was demonstrated that the wormwood extract obtained from stems showed a slightly increased antioxidant effect compared to the leaves extract. Looking at the wormwood extracts obtained from leaves it was showed that with increasing concentration, antioxidant activity increases. Our results are in agreement with those obtained by Kim and co-workers [32]. Lee and co-workers reported an antiradical activity of *A. absinthium* roots (IC_50_ = 271.34 μg/mL*)*. They also found for *A. annua* high IC_50_ values of antiradical activities (IC_50_ = 190.54 μg/mL for leaves extract and IC_50_ = 22.90 μg/mL for stems extracts). Comparable to our results are the values obtained by the same group of authors for *A. capillaries* leaves extract (IC_50_ = 448.15 μg/mL) and *A. selengensis* stems extract (IC_50_ = 411.85 μg/mL) [33]. 

Slightly higher values regarding the radical-scavenging activities of wormwood extract were reported by Mahmoudi et al. [34]. These authors found IC_50_ values of 612 μg/mL for the DPPH radical-scavenging activity, in case of an *A. absinthium* extract obtained from aerial parts collected during flowering stage.

### 3.3. Bioactivity

The in vitro studies performed in this paper demonstrate that *A. absinthium* leaves and stems extracts exhibited a dose dependent anti-migratory and cytotoxic effect on the two tumor cell lines, melanoma and breast adenocarcinoma cells. An important aspect is represented by the data obtained for non-tumor cells (human keratinocytes), where *A. absinthium* extracts had no significant cytotoxic effect at 24 and 48 h post-stimulation; at 72 h after stimulation the cytotoxic effect was lower compared to the effect obtained on cancer cells. The differences between the cytotoxic activity on tumor and non-tumor cells suggest that *A. absinthium* extracts affect more the cancer cells.

Our results regarding the fact that wormwood has a more potent effect on cancer cells, are in accordance with the data found in the literature, where Koyuncu [35] evaluated the anticancer activity of *A. absinthium* methanolic extract on DLD-1 colon cancer cells, ECC-1 endometrium cancer cells and HEK-293 embryonic kidney cells and indicated that it had a cytotoxic effect on the cancer cells while showing low cytotoxicity on the kidney cells. Gordanian et al. [36] tested the effect of five *Artemisia* species (*A. absinthium*, *A. vulgaris*, *A. incana*, *A. fragrans* and *A. spicigera*) harvested from Iran against MCF7 breast adenocarcinoma and HEK-293 embryonic kidney cells. *A. absinthium* L. methanolic extract displayed a stronger cytotoxic effect against MCF7 cells than on HEK-293 cells [36]. The same authors evaluated the anti-cancer effect of various *A. absinthium* L. extracts obtained from different parts of the species—flower, leaf, stem or root and demonstrated that the most potent decrease in MCF7 cells viability was obtained after stimulation with the methanolic extract obtained from flowers, followed by the leaf, stem and finally the root methanolic extract [36]. The present study demonstrated strong cytotoxic and anti-migratory activities against both A375 and MCF7 cancer cells after stimulation with *A. absinthium* stems ethanolic extract.

Another aim of the present study was to determine the degree of selectivity of *A. absinthium* leaves and stems extracts. Even though the results obtained in the cytotoxicity assay evidenced that *A. absinthium* extracts affect more the melanoma and breast adenocarcinoma cells rather than the human keratinocytes, the tested samples did not show a selective effect on the cancer cells after the determination of SI; the values obtained were lower than 2. It is considered that the higher the SI is, the more potent and differential a sample is [37]. According to Peña-Morán et al. [38] for a compound to be considered selective the SI value must be higher than 10. The same authors stated that compounds that have the SI between 1 and 10 are considered non-selective [38].

Shafi and co-workers [39] proved that *A. absinthium* methanolic extract obtained from the aerial parts inhibits cell proliferation and triggers apoptosis in two breast cancer cells, namely MDA-MB-231 and MCF7 cells. In a recent study it was shown that *A. absinthium* ethanolic extract inhibited BEL-7404 human hepatoma and H22 mouse hepatoma cells growth and induced apoptosis without affecting the normal hepatic cells [40].

The TPA model of ear inflammation is a very useful tool for assessing anti-inflammatory compounds. When applied to mice ear skin, TPA provokes an influx of mast cells that release mediators responsible for an increased vascular permeability and for neutrophils infiltration [41]. This is consistent with our results, where topical application of TPA led to a massive pro-inflammatory response in mouse ear such as interstitial edema, diffuse inflammation, exocytosis and micro-abscesses.

The group treated with *A. absinthium* leaves extract showed a mild anti-inflammatory effect. By contrast, topical application of *A. absinthium* stems extract elicited a noticeable anti-inflammatory effect in TPA-induced ear inflammation in mice. Histological evaluation showed that *A. absinthium* stems extract inhibited infiltration of neutrophils into the site of inflammation as there was a mild infiltrate with neutrophils at the dermis level, without micro-abscesses, exocytosis or leucocytes margination.

## 4. Materials and Methods

### 4.1. Chemicals and Reagents

Ethanol 80% (*v*/*v*) and distilled water, purchased from Chemical Company SA (Iasi, Romania) were used to obtain the wormwood extracts (leaves and stems). In order to investigate the antioxidant effect, 2,2-diphenyl-1-picrylhydrazyl (DPPH, Batch No: # STBF5255V, purchased from Sigma Aldrich, Steinheim, Germany) was used The ascorbic acid used as etalon for determination of antioxidant effect, was acquired from Lach-Ner Company (Prague, Czech Republic). For the determination of total phenolic content, were used gallic acid 98% and Na_2_CO_3_ 99%, acquired from Roth (Dautphetal, Germany) and Folin-Ciocalteu reagent, purchased from Merck (Darmstadt, Germany). For the determination of total flavonoid content, were used NaNO_2_ purchased from Merck; AlCl_3_ 98% acquired from Roth and NaOH pellets, acquired from ChimReactiv SRL (Bucharest, Romania). (+)-Cathechin hydrate 98% used as standard for the determination of flavonoid content was acquired from Sigma-Aldrich.

The chemicals used for LC-MS analysis were as follows: methanol (99.9% purity) and acetic acid (99.9% purity), purchased from Merck. Standard polyphenolic compounds were: gentisic acid, chlorogenic acid, caffeic acid, *p*-coumaric acid, isoquercitrin, rutin, quercitrin, luteolin, and apigenin were purchased from Sigma-Aldrich. Ultrapure deionized water was provided by a MiliQ system Milli-Q^®^ Integral Water Purification System (Merck Millipore, Darmstadt, Germany).

The chemicals used for cell culture were purchased from Sigma-Aldrich (Taufkirchen, Germany) and Thermo Fisher Scientific (Boston, MA, USA). TPA (12-*O*-tetradecanoylphorbol-13-acetate) was acquired from Sigma-Aldrich. For the experimental protocol the substance was dissolved in acetone at a concentration of 8 × 10^−4^ M. Acetone (analytical purity of 99.92%) was purchased from ChimReactiv SRL.

### 4.2. Cell Lines

A375-human melanoma cell line (ATCC^®^ CRL-1619 ^TM^) and MCF7—human breast adenocarcinoma cell line (ATCC^®^ HTB-22^TM^) were purchased from the American Type Culture Collection (ATCC, Manassas, VA, USA); HaCaT—human keratinocytes were kindly provided by the University of Debrecen (Debrecen, Hungary). A375 and HaCaT cells were cultured in high glucose Dulbecco’s Modified Eagle’s Medium (DMEM; Sigma-Aldrich); MCF7 cells were cultured in Eagle’s Minimum Essential Medium (EMEM; ATCC). Each cell line was supplemented with 10% fetal bovine serum (FBS; Gibco, Thermo Fisher Scientific) and 1% antibiotic mixture (Penicillin/Streptomycin—Pen/Strep, 10,000 IU/mL; Sigma-Aldrich). The cells were kept under standard conditions (37 °C and humidified atmosphere containing 5% CO_2_).

### 4.3. Plant Material

*A. absinthium* leaves and stems were collected in June 2018 from Vâlcea county (Romania; coordinates: 44°59′17.46″ N, 23°52′5.59″ E) and identified by Professor DS Antal from the Department of Pharmaceutical Botany. A voucher specimen (no. MA_AA1) was deposited at the Herbarium of the Faculty of Pharmacy, Timisoara. After collection, the samples were dried in a plant dryer at room temperature and conserved in a desiccator at 20 °C in darkness until further uses.

### 4.4. Preparation of A. absinthium L. Ethanolic Extracts

Ethanolic extracts from *A. absinthium* leaves and stems were obtained according to the method of Mau et al., slightly modified [42]. Procedures were as follows: 1 g of dried and ground sample (leaves/stem) was mixed with 60 mL ethanol 80% and sonicated for 1 h at room temperature (amplitude A = 50% and cycle C = 0.5) with an UP200S Ultrasonic Homogenizer from Hielscher (Wanaque, NJ, USA).

**Figure 6:**
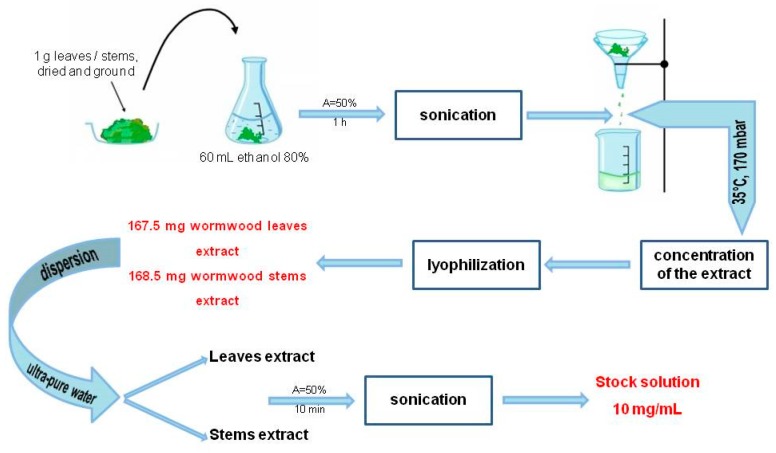
Schematic protocol of *A. absinthium* L. ethanolic extracts preparation.

After sonication, both samples were filtered through a Whatman no. 4 filter paper and concentrated under reduced pressure at 35 °C using a rotary evaporator. Each dried crude extract was subsequently lyophilized and stored at −4 °C. Lyophilized extracts were further employed for the preparation of aqueous stock solutions (concentration 10 mg/mL). Dispersion in ultrapure water was enhanced by sonication (50% amplitude) for 10 min; finally the obtained stock solutions were filtered. The schematic protocol is depicted in Figure 6.

### 4.5. Physico-Chemical Characterization

Thermal behavior of the two dried lyophilized extracts was studied using a STA 449C instrument (Netzsch, Selb, Germany) in air atmosphere at a flow rate of 20 mL·min^−1^. The TG/DSC curves were recorded in the range 25–1000 °C with a heating rate of 10 K·min^−1^, using alumina crucibles.

The FT-IR spectra of the dried lyophilized of *A. absinthium* leaves and stems extracts were obtained using a Prestige-21 spectrometer (Shimadzu, Duisburg, Germany) at room temperature conditions. The spectral region ranged from 4000–400 cm^−1^ using KBr pellets and a resolution of 4 cm^−1^.

LC-MS experiments were conducted on an 6120 LC-MS analytical system from Agilent (Santa Clara, CA, USA) consisting of 1260 Infinity HPLC equipped with G1322A degasser, G1311B cuaternary pump, G1316A column thermostat, G1365C MWD detector and G7129A autosampler coupled with a Quadrupolar (Q) mass spectrometer and electrospray ionization source (ESI). LC-MS is connected to a PC computer running the OpenLAB CDS ChemStation Workstation software.

The samples preparation for LC-MS analysis were homogenized with a WisdVM-10vortex mixer (Witeg Labortechnik, Wertheim, Baden-Württemberg, Germany) and centrifuged for 2 min at 10,000 rpm in a ThermoMicro CL17microcentrifuge (Thermo Fisher Scientific). The supernatant was collected and submitted to LC-MS analysis. Polyphenolic compounds were separated on a reverse phase Zorbax Eclipse Plus C18 column (3.0 mm × 100 mm × 3.5 µm). Gradient elution with a mixture of 0.1% acetic acid and methanol as mobile phase consists was performed: the first 5 min 5% methanol, until 38 min in gradient elution up to 42% methanol, proportion kept until 41 min and ending with 5% methanol for 1 more minute as described before [43]. The injection volume was 10 µL, the flow rate was 1 mL/min and the column temperature 40 °C. UV detection was conducted at 330 and 370 nm. MS detection was achieved by electrospray ionization (ESI) in the single ion monitoring mode (SIM) simultaneous for all screened compounds. All mass spectra were recorded in the negative ion mode. Capillary voltage was set at 3500 V, the dry gas flow was 12 L/min at 350 °C, the nebulizer pressure was kept at 55 psi and the fragmentor was set at 70. For the quantification of polyphenolic compounds calibration curves were conducted by the external standard method in the 0.05–2 µg/mL range for a six-point plot for each compound. The *m*/*z* scale of the mass spectrum was calibrated by use of an external calibration standard ESI Tuning Mix from Agilent.

### 4.6. Antioxidant Activity Assay

The antioxidant activity of *A. absinthium* extracts was determined by DPPH (2,2-diphenyl-1-picrylhydrazyl) free-radical scavenging assay. The electron donation ability of wormwood leaves and stems extracts was measured spectrophotometrically, leading to the yellow coloration of the initially purple-colored DPPH solution, according to the method of Manzoco et al. [44]. To 0.5 mL ethanolic extract of *A. absinthium* leaves/stems extracts (10–0.5 mg/mL), 2 mL solvent (ultrapure water) and 0.5 mL of 1 mM DPPH ethanolic solution were added. During the incubation period, the extracts were kept within the UV/VIS Spectrophotometer (T 70 type, from PG Instruments Ltd., Leicestershire, United Kingdom) where the absorbance of the samples was read continuously (for 10 min) against a blank at 516 nm. The results were expressed in comparison to an ethanol solution of ascorbic acid 0.1 mg/mL. The inhibition percentage of free radical DPPH, expressed as (IP%), was calculated with the formula below:

(1) $$IP\;\%\;=\left[\frac{A_{DPPH}-A_{sample}}{A_{DPPH}}\right]*100$$

where: $A_{DPPH}$ is the absorbance of free radical DPPH (blank), measured at 516 without sample and $A_{sample}$ is the absorbance of each sample in the presence of DPPH radical.

The half maximal inhibitory concentration (IC_50_) was calculated by linear regression analysis curve plotting between inhibition percentage (IP%) and concentration of the ethanolic wormwood extracts. 

### 4.7. Determination of Total Phenolic Content and Total Flavonoid Content of A. Absinthium

The total phenolic content of the ethanolic extracts from wormwood leaves and stems was evaluated using the Folin-Ciocalteu method, slightly modified [45]. The method was briefly the following: 0.5 mL of extract solutions (1 mg/mL) were mixed with 2.5 mL Folin-Ciocalteu reagent diluted 1:10. Then 2 mL of 7.5% sodium carbonate solution were added. Samples were kept in the dark for 90 min, then the absorbance was read versus blank at 750 nm using a UviLine 9400 Spectrophotometer from SI Analytics (Deutschland, Germany). The total phenolic content was determined using a gallic acid calibration curve (R^2^ = 0.996). To this end, a gallic acid calibration curve was obtained, using gallic acid solutions of different concentrations (0.0025–0.1 mg/mL). The total phenolic content of the two extracts was calculated as milligrams of gallic acid equivalents (GAE) per gram extract.

The total flavonoid content of the two ethanolic extracts was evaluated using the following method [22]: an aliquot of 1 mg/mL extract solution (250 μL) was mixed with 5% NaNO_2_ (75 μL). After 6 min, 10% AlCl_3_ (150 μL) and 1M NaOH (500 μL) were added. The volume was adjusted to 2.5 mL with distilled water. The absorbance was read at 510 nm versus blank using a UviLine 9400 Spectrophotometer from SI Analytics. The total flavonoid content was calculated using a (+)-Catechin hydrate calibration curve (R^2^ = 0.999) in the range 0.005–0.4 mg/mL. The results were expressed as milligrams catechin equivalents per gram extract (mg CE/g extract).

### 4.8. Alamar Blue Assay–Cell Viability Assessment

The viability was determined using the Alamar blue assay, a technique meant to evaluate the cytotoxic effect caused by different agents. The cells (1 × 10^4^/well) were cultured in 96-well plates, allowed to adhere overnight and then stimulated with different concentrations of the extractive solutions (50, 100, 250, 500 and 1000 µg/mL) for 24, 48 and 72 h. After the stimulation periods, 20 μL of Alamar blue (10% of the volume of cell culture medium—200 μL/well) was added. The cells were incubated for 3h at 37 °C and then the absorbance was measured at two different wavelengths (570 and 600 nm) with an xMark™ Microplate spectrophotometer (BioRad, xMarkTMMicroplate, Serial No. 10578, Tokyo, Japan). The principle of this technique consists of the natural ability of metabolically active cells (viable cells) to reduce resazurin (dark blue compound) to the fluorescent form, resorufin (pink compound with intense fluorescence).

### 4.9. Selectivity-Index

In order to determine the selectivity of *A. absinthium* leaves and stems extracts, the selectivity index was calculated according to the following formula [38,46]:

(2) $$Selectivity\;index\;\left(SI\right)\;=\;\frac{IC_{50\;\left[non-malignant\;HaCaT\right]}}{IC_{50\;\left[tumor\;cell\;line\right]}}$$

### 4.10. Scratch Assay–Assessment of the Anti-Migratory Potential

This method is an in vitro technique used to determine a possible inhibitory effect of *A absinthium* extractive solutions on the migration and invasion capacity of the tumor cell lines (A375 and MCF7 cells) and of the non-tumor cells (HaCaT). The protocol was applied as previously described in the literature [47]. Briefly, 2 × 10^5^ cells/well were cultured in 12-well plates until 90% confluence was reached. Using a sterile pipette tip scratches were drawn on well-defined zones of the cells monolayer. The detached cells resulted from the procedure were removed by washing with phosphate-buffered saline (PBS) prior to stimulation. The cells were stimulated with different concentrations of the extractive solutions (50, 100, 250, 500 and 1000 µg/mL). Images of the cells in culture were taken at 0 h and 24 h and compared with the control cells (no stimulation). Pictures were taken with Olympus IX73 inverted microscope provided with DP74 camera (Olympus, Tokyo, Japan) and the analysis of the cell growth was performed with cell Sense Dimension software. The migration percentage was calculated according to the formula previously described by Felice et al. [48]:

(3) $$Scratch\;closure\;rate\;=\;\left[\frac{A_{t_{0}}-A_{t}}{A_{t_{0}}}\right]*100$$

where: $A_{t_{0}}$ is the scratch area at time 0; $A_{t}$ is the scratch area at 24 h.

### 4.11. In Vivo TPA-Induced Ear Inflammation Protocol

In order to evaluate the effect of *A. absinthium* leaves and stems extracts on ear inflammation were performed *in vivo* experiment using SKH1 female mice (n = 3), 6 months old (weigh = 29.70–32.80 g); purchased from Charles River Laboratory (Budapest, Hungary). The mice were kept in standard conditions: a 20–24 °C temperature, humidity between 45–65%, a 12-h light/dark cycle, food *ad libitum* with free access to water, as recommended by the European Directive 2010/63/EU and the national law 43/2014. The protocol used for the euthanasia of the animals followed the Guidelines described by the American Veterinary Medical Association (AVMA) for the Euthanasia of Animals (2013 Edition). The Bioethical Committee of “Victor Babes” University of Medicine and Pharmacy Timisoara, Romania, approved the performed experiment.

The experimental model of TPA-induced ear inflammation was performed according to the following protocol: female mice were anesthetized with Isoflurane and the TPA solution was topically applied (20 μL/mouse ear) and they were assigned to the subsequent groups (Table 6).

An electronic caliper was used to measure the diameter and length of mice ears (the results were presented in millimeters) before the TPA application and at the end of the experiment. After 24 h the mice were weighted and after that were anesthetized and sacrificed, the ears were measured and collected for histopathological evaluation.

### 4.12. Histopathological Assessment of Mice Ears

For histopathological analysis, the mice ears were fixed in 10% buffered formalin for 48 h and then they were embedded in paraffin followed the routine automated flow of this procedure. Four µm-thick sections were cut using a Leica Rotary Microtome (Leica Biosystems Nussloch GmbH, Nussloch, Germany) and mounted on glass slides, deparaffinized in xylene and rehydrated. Finally, the samples were stained with the conventional Hematoxylin & Eosin. The slides were examined by two pathologists in a blinded way. Image acquisition and analysis were performed using a Nikon Eclipse E 600 microscope (Nikon Microscopes/Instruments Division, Vienna, Austria) and Lucia G software (Laboratory Imaging, Prague, Czech Republic) for microscopic image analysis.

### 4.13. Statistical Analysis

The statistical program used in the present study was GraphPad Prism 5 (GraphPad Software, San Diego, CA, USA). The data obtained were expressed as mean ± SD. Regarding the in vitro results, comparison among the groups was performed using the one-way ANOVA followed by Dunnett’s multiple comparison test (* *p* < 0.05; ** *p* < 0.01; *** *p* < 0.001).

## 5. Conclusions

Results of this study showed that the ethanolic extracts from *A. absinthium* leaves and stems collected from Southern Romania contain chlorogenic acid, quercitrin, isoquercitrin and rutin. The IR profiles of extracts from leaves and stems of *A. absinthium* revealed the presence of active components with various functional groups (acid, alcohol, alkane, amine, amide, aromatic radicals). Leaves displayed a higher content of total phenolics and flavonoids than stems. Regarding the activity against cancer cells, at the check point of 72 h a significant cytotoxic activity against both MCF7 and A375 cell lines was detected. Wormwood stems extract were slightly more active than leaves extract. In the experimental conditions of our study, both leaves and stems extracts showed dose-dependent anti-migratory potential, MCF7 being the most sensitive cell line. Moreover, stems extract elicited a stronger anti-migratory activity. In an *in vivo* model of inflammation, the topical application of stems extract led to a noticeable anti-inflammatory effect, while the activity of the leaves extract was milder. Overall, A. *absinthium* from Southern Romania was proved to possess biological activities that can be exploited in further studies.

## Figures and Tables

**Table 1:** Peak values and functional groups of *A. absinthium* leaves and stems extracts in the spectrum.

Characteristic Absorptions [cm^−1^] Leaves Extract/Stems Extract	Functional Group	Bond
3365.78/3381.21	Amines, amide, alcohol	N-H stretching O-H stretch (H-bonded)
2926.01/2924.09; 2852.72	Alkanes	C-H strech
1616.35/1600.92	Amide	N-H bending
1516.05/1506.41	Nitro compounds	N-O asymmetric strech
1456.26	Aromatics	C=C stretch (in ring)
1398.39/1384.89	Alkanes	-C-H bending
1338.60/-	Amines	C-N strech
1265.30/1269.16; 1238.30	Acids	C-O strech
1068.56/1068.56; 1124.50	Alcohols	C-O stretch
866.04/912.33	Alkenes	=C-H bending
817.82/817.82	Alkenes	=C-H bending
769.60/767.67	Alkenes	=C-H bending
655.80/657.73	Alkenes	=C-H bending
617.22/611.43	Alkenes	=C-H bending
534.28/534.28	Alkenes	=C-H bending

**Table 2:** Total phenolic and flavonoid contents of the ethanolic wormwood extracts.

Extract	Total Phenolic Content (mg GAE/g Extract)	Total Flavonoid Content (mg CE/g Extract)
Leaves extract	54.68 ± 1.93	43.08 ± 2.47
Stems extract	44.15 ± 1.12	34.14 ± 2.16

**Table 3:** Polyphenolic content of extracts analysed by LC-MS.

	Compound Name	Rt (min)	[M − H^+^]^+^ (*m*/*z*)	*A. absinthium* Leaves (µg/mg d.w.)	*A. absinthium* Stems (µg/mg d.w.)
1.	Gentisic acid	2.67	153	ND	NQ
2.	Chlorogenic acid	6.45	353	1.94	2.03
3.	Caffeic acid	6.97	179	NQ	NQ
4.	*p*-Coumaric acid	10.56	163	NQ	ND
5.	Isoquercitrin	22.50	463	0.04	0.07
6.	Rutin	23.01	609	0.08	0.55
7.	Quercitrin	26.18	447	0.11	0.05
8.	Luteolin	32.78	285	NQ	ND
9.	Apigenin	36.91	269	NQ	ND

Notes: ND—not detected, below the limit of detection; NQ—not quantified, below the limit of quantification.

**Table 4:** The inhibition percentage of *A. absinthium* extracts obtained from leaves and stems, as compared to the inhibition percentage of ascorbic acid ^1^.

Ascorbic Acid		*A. absinthium* Leaves Extract		*A. absinthium* Stems Extract	
Concentration [mg/mL]	% Inhibition	Concentration [mg/mL]	% Inhibition	Concentration [mg/mL]	% Inhibition
0.1	94.88 ± 0.029	2	53.11 ± 0.014	2	55.77 ± 0.054
0.08	95.47 ± 0.001	1.4	49.47 ± 0.015	1.4	56.84 ± 0.026
0.06	95.06 ± 0.001	0.7	47.32 ± 0.026	0.7	48.79 ± 0.015
0.04	94.85 ± 0.0015	0.2	32.61 ± 0.020	0.2	37.71 ± 0.019
0.02	83.19 ± 0.005	0.1	31.15 ± 0.021	0.1	34.02 ± 0.056

^1^ The results are expressed as average ± SD (*n* = 3).

**Table 5:** IC_50_ values and selectivity of *A. absinthium* leaves and stems extracts (cancer cells vs. non-malignant HaCaT cells—human keratinocytes) at 72 h post-stimulation.

Extract	HaCaT IC_50_ (µg/mL)	A375 IC_50_ (µg/mL)	MCF7 IC_50_ (µg/mL)	SI *
***A. absinthium* leaves**	397.7 ± 7.2	295.4 ± 7.1 -	- 250.6 ± 6.3	1.35 1.59
***A. absinthium* stems**	361.8 ± 9.3	312 ± 3.4 -	- 246.8 ± 7.2	1.16 1.47

* Selectivity index (SI) is calculated as the ratio between the IC_50_ values; IC_50 [non-malignant HaCaT]_/IC_50 [tumor cell line]_. The data represent the mean values ± SD of three independent experiments.

**Table 6:** In vivo experimental design for the local acute inflammation model.

Group No.	Group Name	Description
1	Control	With no intervention
2	Control + Acetone	Acetone (solvent for TPA)—20 μL/mouse ear
3	TPA	TPA solution (20 μL/mouse ear)
4	TPA + Indomethacin	Indomethacine cream (4%) was topically applied after the TPA solution
5	TPA + *A. absinthium* L. leaves extract	*A. absinthium* leaves extract (~2%) was topically applied after the TPA solution
6	TPA + *A. absinthium* L. stems extract	*A. absinthium* stems extract (~2%) was topically applied after the TPA solution

## Supplementary Materials

The supplementary materials are available online.

## Abbreviations

LC-MS	Liquid chromatography–mass spectrometry
TG-DSC	Thermogravimetry-differential scanning calorimeter analysis
FT-IR	Fourier-transform infrared spectroscopy
Rt	Retention time
DPPH	2,2-Diphenyl-1-picrylhydrazyl
AOA	Antioxidant activity
IC_50_	Maximal inhibitory concentration
IP	Inhibition percentage
HaCaT	Human keratinocytes
MCF7	Human breast adenocarcinoma cell line
PBS	Phosphate-buffered saline
A375	Human melanoma cell line
ATCC	American Type Culture Collection
DMEM	Dulbecco’s Modified Eagle’s Medium
EMEM	Eagle’s Minimum Essential Medium
FBS	Fetal bovine serum
SD	Standard deviation
TPA	12-*O*-Tetradecanoylphorbol-13-acetate
ROS	Reactive oxygen species
MS	Mass spectroscopy
ESI	Electrospray ionization
SIM	Single ion monitoring mode
SI	Selectivity index
mg GAE	Milligrams of gallic equivalents
mg CE	Milligrams cathechin equivalents
