# Supplementary figures and images for: Romanian Wormwood (Artemisia absinthium L.): Physicochemical and Nutraceutical Screening
Source: Molecules. 2019 Aug 25;24(17):3087. doi: 10.3390/molecules24173087 (PMC6749517; doi:10.3390/molecules24173087)

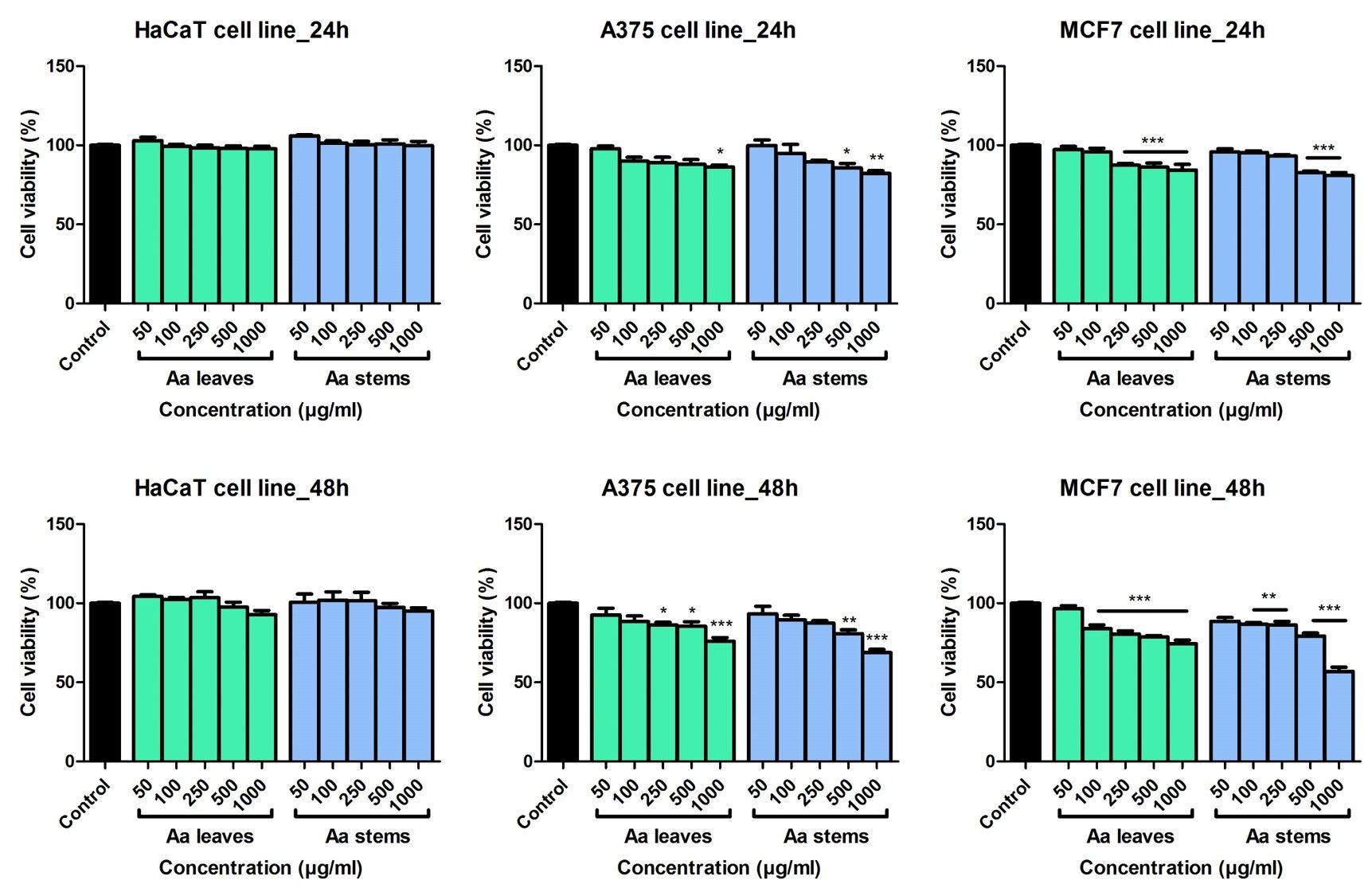

Supplement: Supplementary file 1 [file molecules-24-03087-s001.zip › Supplementary_files/Supplementary figure 1.jpg]

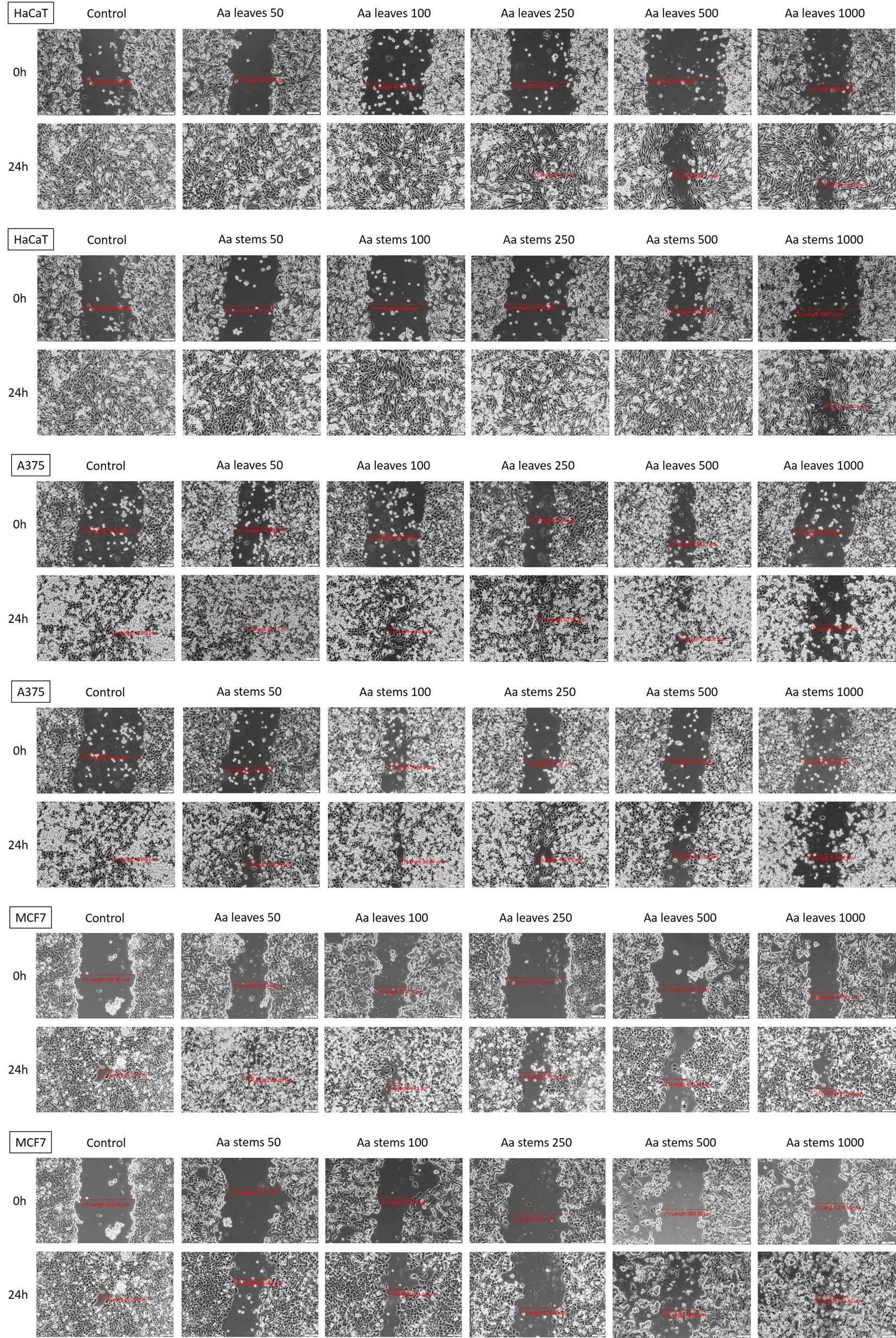

Supplement: Supplementary file 1 [file molecules-24-03087-s001.zip › Supplementary_files/Supplementary figure 2.jpg]
